# Editome profiling and cross-cohort validation reveal A-to-I RNA editing dysregulation in the hippocampus and prefrontal cortex of sepsis patients

**DOI:** 10.3389/fpsyt.2025.1742710

**Published:** 2026-03-03

**Authors:** Jie Shen, Jia-Qi Pan, Tian-Yue Yu, Le-Wen Liu, Yu-Nuo Li, Yun-Yun Jin, Minmin Zhu

**Affiliations:** 1 Department of Anesthesiology and Pain Medicine, Jiangnan University Medical Center, Wuxi No.2 Hospital, Wuxi, China; 2 Laboratory of Genomic and Precision Medicine, Wuxi School of Medicine, Jiangnan University, Wuxi, Jiangsu, China

**Keywords:** A-to-I RNA editing, mitochondrialantiviral-signaling protein, sepsis, the hippocampus, the prefrontal cortex

## Abstract

**Introduction:**

Sepsis is a severe systemic inflammatory response to infection, potentially resulting in serious neurological complications. Adenosine-to-inosine (A-to-I) RNA editing is a critical epitranscriptomic process, yet its clinical involvement in different brain regions during sepsis remains to be elucidated.

**Methods:**

We conducted a comprehensive editome analysis and experimental validation to characterize A-to-I RNA editing in hippocampal and prefrontal cortical tissues from a sepsis cohort, as well as in brain tissues from a validation cohort.

**Results:**

Our findings revealed diverse dysregulation of RNA editing in these brain regions, with a substantial reduction in average RNA editing levels in the hippocampus but not in the prefrontal cortex. Moreover, cross-cohort and cross-tissue analysis identified eight key genes with dysregulated RNA editing during sepsis, particularly mitochondrial antiviral-signaling protein (*MAVS*), whose RNA editing was significantly downregulated. *ADAR* knockout or knockdown in multiple cell lines reduced both *MAVS* RNA editing and expression, confirming *MAVS* as a target of ADAR-mediated A-to-I RNA editing. Moreover, human vascular endothelial cells stimulated with LPS for 2 hours exhibited downregulated *MAVS* RNA editing, consistent with findings in sepsis patients, as well as reduced *ADAR* and *MAVS* expression, suggesting a potential role for ADAR-mediated RNA editing in regulating *MAVS* expression during inflammation. Consistently, *ADAR* and *MAVS* expression showed a positive correlation in both brain regions (Spearman’s ρ > 0.49, *P* < 1 × 10^-7^).

**Discussion:**

Our results thus provide new insight into the importance of the clinical epitranscriptomic landscape in different brain regions during sepsis and warrant further investigation into therapeutic strategies to mitigate cognitive impairment in sepsis.

## Introduction

Sepsis is a life-threatening systemic inflammatory response to infection that frequently causes multiple organ dysfunction (1). One of the most common complications of sepsis is sepsis-associated encephalopathy (SAE), a diffuse cerebral dysfunction that occurs without a direct central nervous system infection (2). SAE manifests acutely as delirium and long-term as cognitive deficits, anxiety, depression, and increased risk of neurodegenerative diseases in survivors (3–5). Studies have shown that essential brain regions involved in higher cognitive functions—especially the hippocampus, which is crucial for memory and spatial navigation, and the prefrontal cortex, vital for executive control, decision-making, and emotional regulation—are significantly impacted during SAE (6).

Several mechanisms have been implicated in SAE, such as neuroinflammation (7), microglial activation (8), blood–brain barrier disruption (9), and mitochondrial dysfunction (10). Nevertheless, the epitranscriptomic landscape underlying sepsis-induced brain dysfunction remains largely unexplored. Adenosine-to-inosine (A-to-I) RNA editing is a post-transcriptional RNA epigenetic alteration mediated by adenosine deaminase acting on RNA (ADAR) enzymes (11), resulting in amino acid substitutions, modulation of splicing patterns, and altered miRNA targeting (12). A-to-I RNA editing is highly enriched in the brain with region- and cell type-specific patterns, highlighting its essential role in the development and function of the central nervous system (13). Dysregulation of RNA editing has been implicated in various neuropsychiatric and neurodegenerative disorders, including schizophrenia, major depression, autism spectrum disorder, and Alzheimer’s disease (14–16). In addition, previous studies have reported that inflammation and immune activation can modulate the expression and activity of ADAR enzymes (17, 18), which thereby might trigger alterations in A-to-I RNA editing in SAE. Our previous reports have demonstrated dynamic brain A-to-I RNA editing changes associated with SAE in both patients and mouse models (19–21). However, by far, no prior study has comprehensively profiled A-to-I RNA editing across various brain regions of sepsis patients.

In the current study, we performed an editome analysis of A-to-I RNA editing in post-mortem hippocampus and prefrontal cortex samples from sepsis patients and controls, revealing diverse dysregulation of RNA editing between these brain regions. Importantly, through validation in different brain tissues and independent cohorts, as well as cell experiments, our study identified key genes with dysregulated RNA editing in the septic brain, especially mitochondrial antiviral-signaling protein (MAVS), underlining the role of RNA editing in neuroinflammation and neuronal dysfunctions during the disease. Our findings thus provide new insight into novel epitranscriptomic mechanisms contributing to SAE and may offer new avenues for biomarker discovery or therapeutic intervention.

## Materials and methods

### RNA-seq datasets

RNA-Seq reads were collected from the NCBI Gene Expression Omnibus (GEO) database. The discovery cohort dataset (GEO accession GSE237861) contained postmortem hippocampal and prefrontal cortex samples from seven sepsis patients and seven controls (22). Another sepsis cohort dataset included brain samples from twelve sepsis patients and twelve controls (23). In addition, RNA-Seq datasets of wildtype (WT) and *ADAR*-knockout human HEK293T embryonic kidney cells (GSE99249) (24), as well as human lung carcinoma A549 cells (GSE147487) (25) and human lymphoblastoid GM19238 cells (GSE52834) treated with *ADAR* siRNA and scrambled negative control (NC) siRNA, were used to validate selected RNA editing sites. The RNA extraction, library construction, and sequencing procedure were described in the original studies.

### Read mapping and processing

The sequencing data were then processed as previously described (26). In brief, sequencing read quality was assessed using FASTQC, and processed using FASTP software to remove reads with low quality if detected (27). Clean data were then mapped to the UCSC human hg38 genome sequence using RNA STAR (version 2.7.0e) (28). Multiple-mapped and duplicated reads were filtered out using SAMtools (version 1.9) (29). Base quality score was recalibrated using GATK v.4.1.3 (30).

### Identification of RNA editing events

Identification of RNA editing events was conducted using a previously described bioinformatic pipeline (26). Single-nucleotide variants (SNVs) were called using VarScan (version 2.4.4) (31) with parameters: base quality ≥ 25, alternative allele depth ≥ 2, total sequencing depth ≥ 10, and alternative allele frequency (AAF) ≥ 1%. SNV annotation was performed using the Ensembl Variant Effect Predictor (VEP) (32). High-confidence RNA editing candidate sites were defined as: 1) annotated as known editing sites in the REDIportal V2.0 database (33); 2) A-to-G SNVs in the coding strand passing a set of filters for quality control described in our previous study (26). The post-filtering high-confidence A-to-G SNVs were subjected to subsequent data analysis.

### Cell culture and lipopolysaccharide treatment

Human retinal microvascular endothelial cells (HRMECs) were from the American Type Culture Collection (ATCC, Manassas, VA) and cultured in Dulbecco’s Modified Eagle Medium (DMEM) (high glucose) supplemented with 10% fetal bovine serum. For LPS treatment, HRMECs were cultured in medium containing LPS (200 ng/mL) (Solarbio, L8880) for 2 hours.

### Transfection of siRNAs

Oligonucleotides of the *ADAR* siRNA and negative control (NC) siRNA listed in **Supplementary Table S1** were purchased from GenePharma (Suzhou, China) and transfected into HRMECs using Lipofectamine 3000 (Thermo Fisher). Cells were collected 48 hours post-transfection for total RNA extraction.

### RNA extraction and reverse transcription

Total RNA extraction was performed using the TIANGEN RNAprep Pure Tissue Kit (DP419), and RNA quality was assessed using the Agilent 2100 Bioanalyzer (Agilent Technologies). Reverse transcription of RNA was conducted using HiScript III RT SuperMix (Vazyme Biotech) for cDNA synthesis.

### Polymerase chain reaction and sanger sequencing

The *MAVS* 3′-UTR was amplified from cDNA by PCR. Real-time quantitative PCR (qPCR) was conducted on a LightCycler^®^ 480 II (Roche) with AceQ qPCR Master Mix (Vazyme Biotech). RNA expression was quantified using the 2*^-ΔΔCt^* method and normalized to the housekeeping gene beta-actin (*ACTB*). The primers used are listed in **Supplementary Table S1**.

### Prediction of RNA-binding protein binding sites

The web tool RBPmap (https://rbpmap.technion.ac.il/) was used to predict RBP binding sites that overlap with RNA editing sites, and results were visualized using the wordcloud package in R.

### Expression dataset of the human brain

The expression dataset of *ADAR* and *MAVS* in the human brain was retrieved from the Genotype-Tissue Expression (GTEx) Project (https://www.gtexportal.org/) and cProSite database (https://cprosite.ccr.cancer.gov/).

### Statistical analysis

The differences in RNA editing and expression levels between groups were analyzed using a generalized linear model (GLM) and likelihood ratio tests to calculate *P*-values, with the disease status (sepsis or control) as the independent variable. Principal component analysis (PCA) was conducted using R (version 4.4.2) and the autoplot package. Gene expression correlations were analyzed using Spearman’s rank correlation.

## Results

### A-to-I RNA editome profiles of the hippocampus and prefrontal cortex of sepsis patients

**Figure 1:**
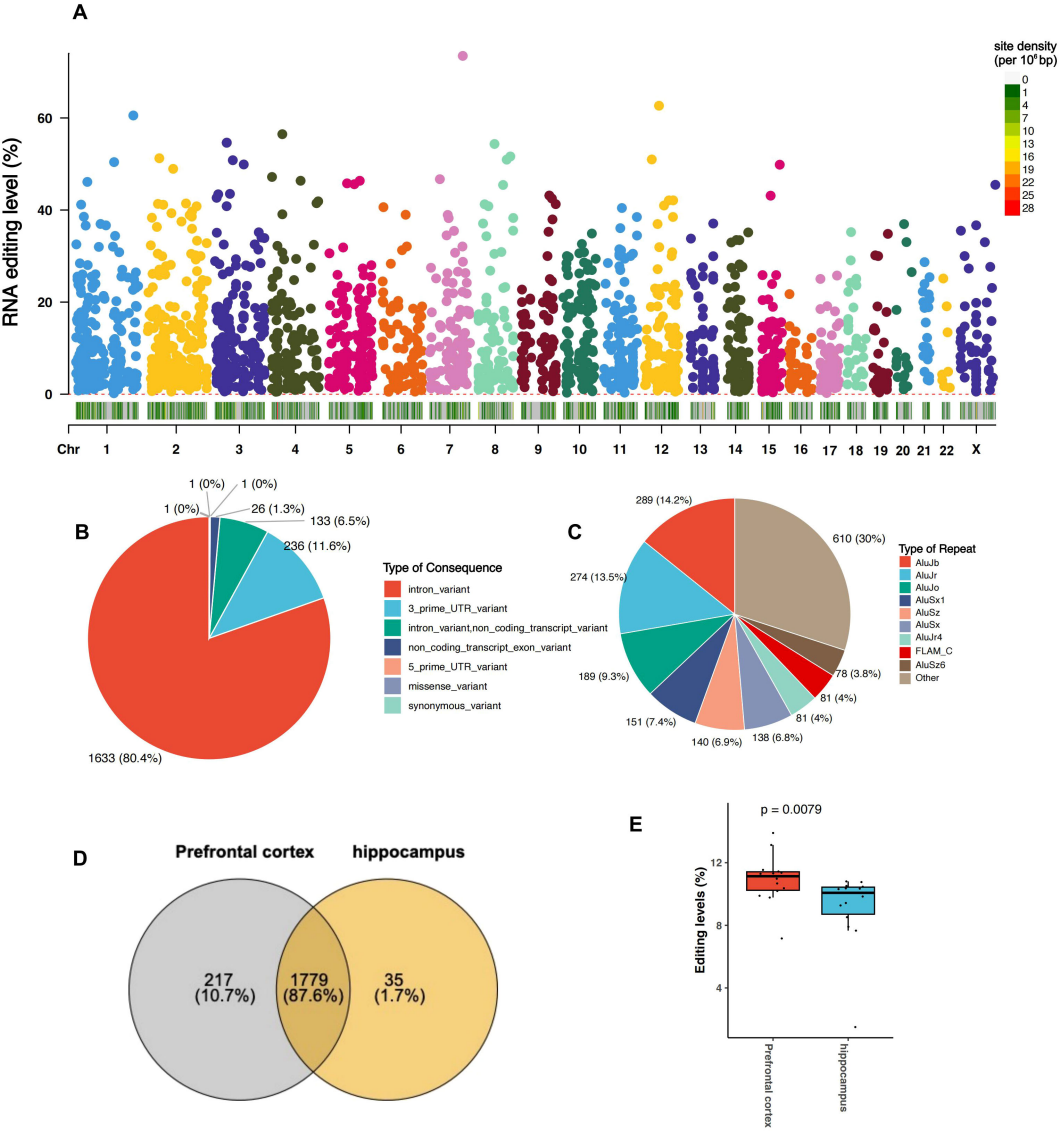
A-to-I RNA editing profiles in the postmortem brain samples from a sepsis cohort. **(A)**. Manhattan plot showing the distribution of the identified A-to-I RNA editing sites across the human genome in the sepsis cohort. Below the chromosomal location, the density of A-to-I RNA editing sites is shown as the number of SNPs within a 1 Mb window. **(B)** Pie chart showing the functional categories of the identified A-to-I RNA editing sites. **(C)** Pie chart showing the distribution of repetitive elements overlapping with the identified A-to-I RNA editing sites. **(D)** Venn plot comparing A-to-I RNA editing sites between the hippocampus and prefrontal cortex. **(E)**.

Our A-to-I RNA editome profiling identified 2031 high-confidence A-to-I RNA editing sites in 1085 genes (**Figure 1A**), 80.4% and 11.6% of which were found in the introns and 3’-untranslated region (3’-UTR) of protein-coding genes (**Figure 1B**, **Supplementary Table S2**). More than 83% of these editing sites were annotated as known sites in the REDIportal database (**Supplementary Table S2**) and most of them were located with Alu elements (**Figure 1C**). As for brain regions, 1,717 and 1,943 high-confidence A-to-I RNA editing sites in 954 and 1054 genes were observed in the hippocampus and prefrontal cortex, respectively (**Figure 1D**, **Supplementary Tables S3**, **S4**). Notably, the transcriptome-wide average RNA editing level was significantly lower in the hippocampus than in the prefrontal cortex (*P*_GLM_ = 0.00079) (**Figure 1E**), suggesting potential differences in the RNA editome between the two brain regions.

### Dysregulation of A-to-I RNA editing in the brain of sepsis patients

**Figure 2:**
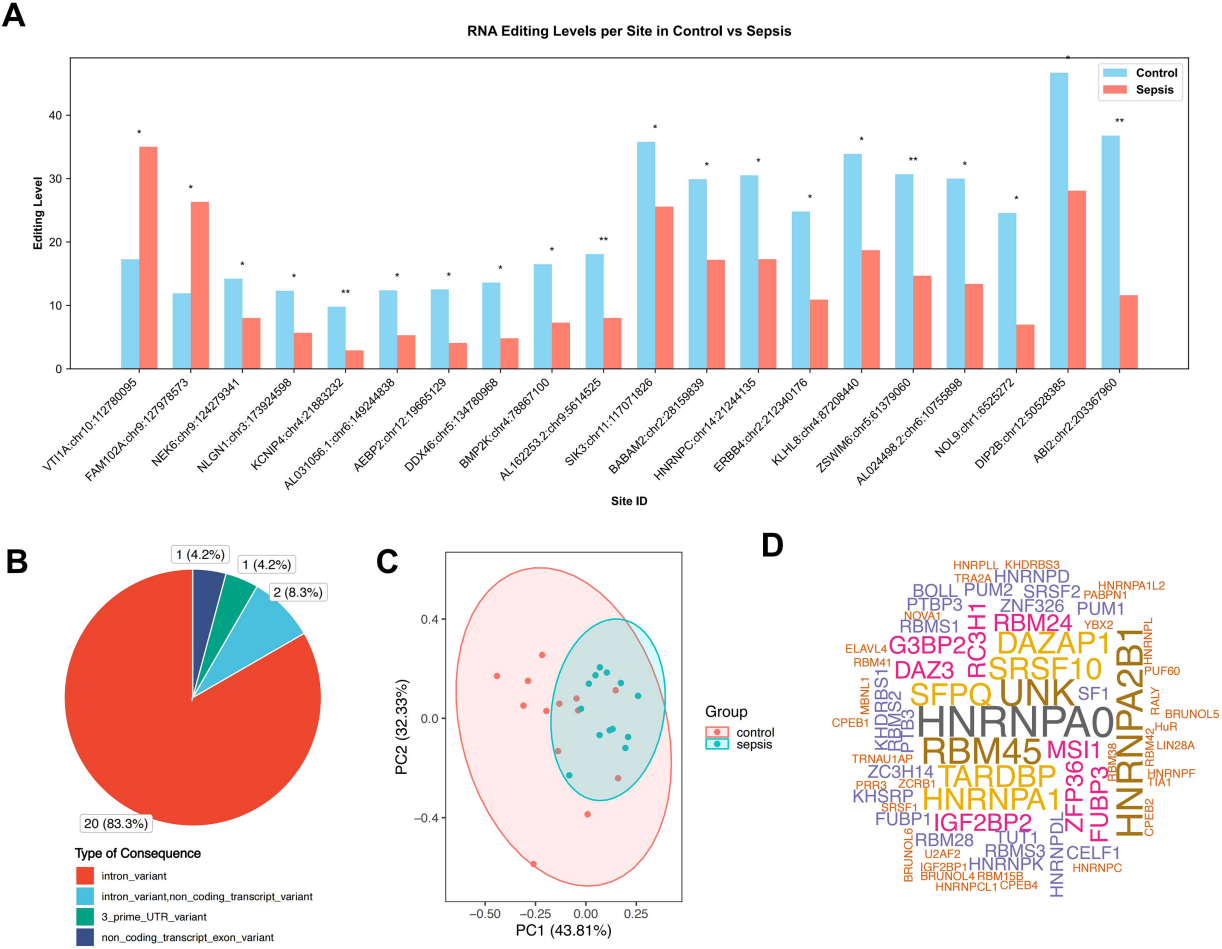
Differential A-to-I RNA editing in the brains between sepsis and controls. **(A)** barplot of the twenty-four differential A-to-I RNA editing sites. **(B)** Pie chart showing the functional categories of the differential A-to-I RNA editing sites in the brain samples. **(C)** PCA results of the differential A-to-I RNA editing sites in the brain samples. **(D)** Cloud plot showing the frequency of RBPs predicted to overlap the differential A-to-I RNA editing sites in the brain samples. *P < 0.05; **P < 0.01.

The differential A-to-I RNA editing sites between sepsis and controls are shown in **Figure 2A**. A total of twenty-four RNA editing sites were found to be differentially edited between sepsis and controls (**Figure 2A**, **Supplementary Table S5**), more than 83% were in the introns of protein-coding genes (**Figure 2B**), which was comparable to the distribution of variant categories in the whole RNA editome. PCA results showed that the PC1 of these differential A-to-I RNA editing events accounted for 43.81% of the total variance between sepsis and control groups (**Figure 2C**). Notably, HNRNPA0 was predicted to be the top one RBP that frequently overlapped with these differential editing sites (**Figure 2D**). Moreover, the editing level of Coatomer subunit gamma-2 (*COPG2*) at chr7:130520321 positively correlated with the gene expression (Spearman *P* = 0.019) and that of Neuroligin 1 (*NLGN1*) at chr3:173924598 negatively correlated with the gene expression (Spearman *P* = 0.024) (**Supplementary Table S6**).

### Region-specific dysregulation of A-to-I RNA editing in the brains of sepsis patients

**Figure 3:**
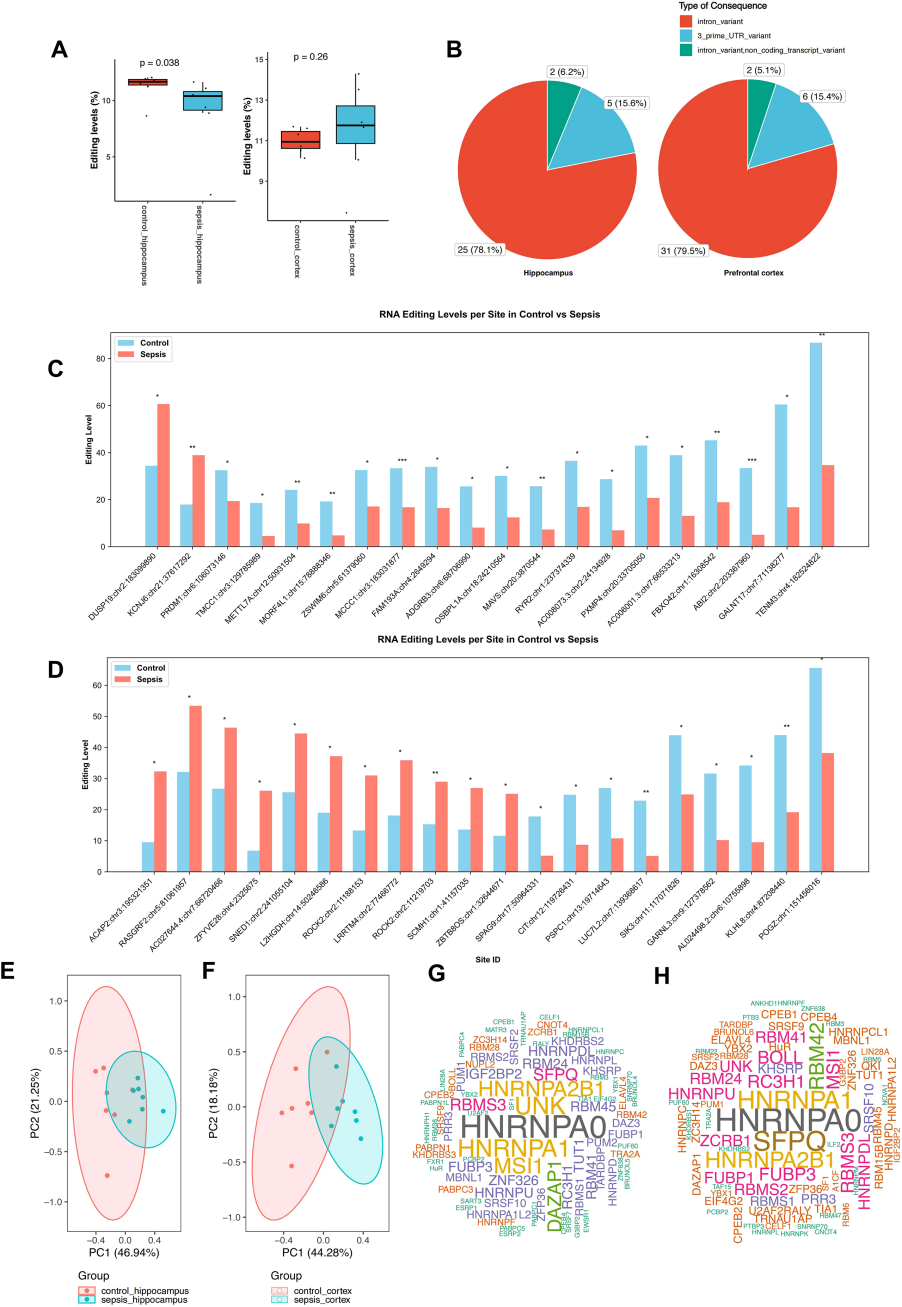
Differential A-to-I RNA editing sites in the hippocampus and the prefrontal cortex between sepsis and controls. **(A)** Comparison of the average RNA editing level between sepsis and controls in the hippocampus and the prefrontal cortex samples. Barplots show the differential A-to-I RNA editing sites between sepsis and controls in the hippocampus **(B)** and the prefrontal cortex samples **(C)**. PCA results of the differential A-to-I RNA editing sites between sepsis and controls in the hippocampus **(D)** and the prefrontal cortex samples **(E)**. Cloud plots show the frequency of RBPs predicted to overlap the differential A-to-I RNA editing sites in the hippocampus **(G)** and the prefrontal cortex samples **(H)**. *P < 0.05; **P < 0.01; ***P < 0.001.

We further analyzed sepsis-associated A-to-I RNA editing profiles in the two different brain regions. Sepsis patients showed a substantial reduction in the hippocampus (GLM test, *P* = 0.038) but not in the prefrontal cortex (**Figure 3A**, **Supplementary Tables S7**, **S8**). Consistently, comparing *ADAR* expression across hippocampal and prefrontal cortex tissues from sepsis and control groups revealed a significant difference between the two brain regions in sepsis (*P* < 0.05, **Supplementary Figure S1**). The distribution of variant categories was similar between the two brain regions (**Figure 3B**). Thirty-two and thirty-nine sites were differentially edited between sepsis and controls (**Figures 3C, D**). PCA results based on these differential A-to-I RNA editing events showed that PC1 accounted for 46.94% and 44.28% of the total variance between sepsis and controls in the two brain regions, respectively (**Figures 3E, F**). HNRNPA0 was consistently the RBP that most frequently overlapped with these differential editing sites in both brain regions (**Figures 3G, H**). In addition, such findings thus revealed significant yet divergent RNA editing patterns associated with sepsis in the hippocampus and prefrontal cortex. The editing level of Oxysterol Binding Protein Like 1A (*OSBPL1A*, chr18:24210564) and Salt-Inducible Kinase 3 (*SIK3*, chr11:117070891) positively correlated with the gene expression in the hippocampus (Spearman *P* = 0.016 and 0.049, respectively) (**Supplementary Table S9**). In contrast, the RNA editing level of Pogo Transposable Element Derived With ZNF Domain (*POGZ*, chr1:151456016), CASP8 And FADD Like Apoptosis Regulator (*CFLAR*, chr2:201164154), LUC7 Like 2, Pre-MRNA Splicing Factor (*LUC7L2*, chr7:139368617), Sperm Associated Antigen 9 (*SPAG9*, chr17:50964331), and Tet Methylcytosine Dioxygenase 3 (*TET3*, chr2:74085081) positively correlated with the gene expression (Spearman *P <* 0.05), whereas WBP1L:chr10:102773778 negatively correlated with the gene expression (Spearman *P* = 0.042) (**Supplementary Table S10**).

### Cross-validation of key genes differentially edited in the brains of sepsis patients

**Figure 4:**
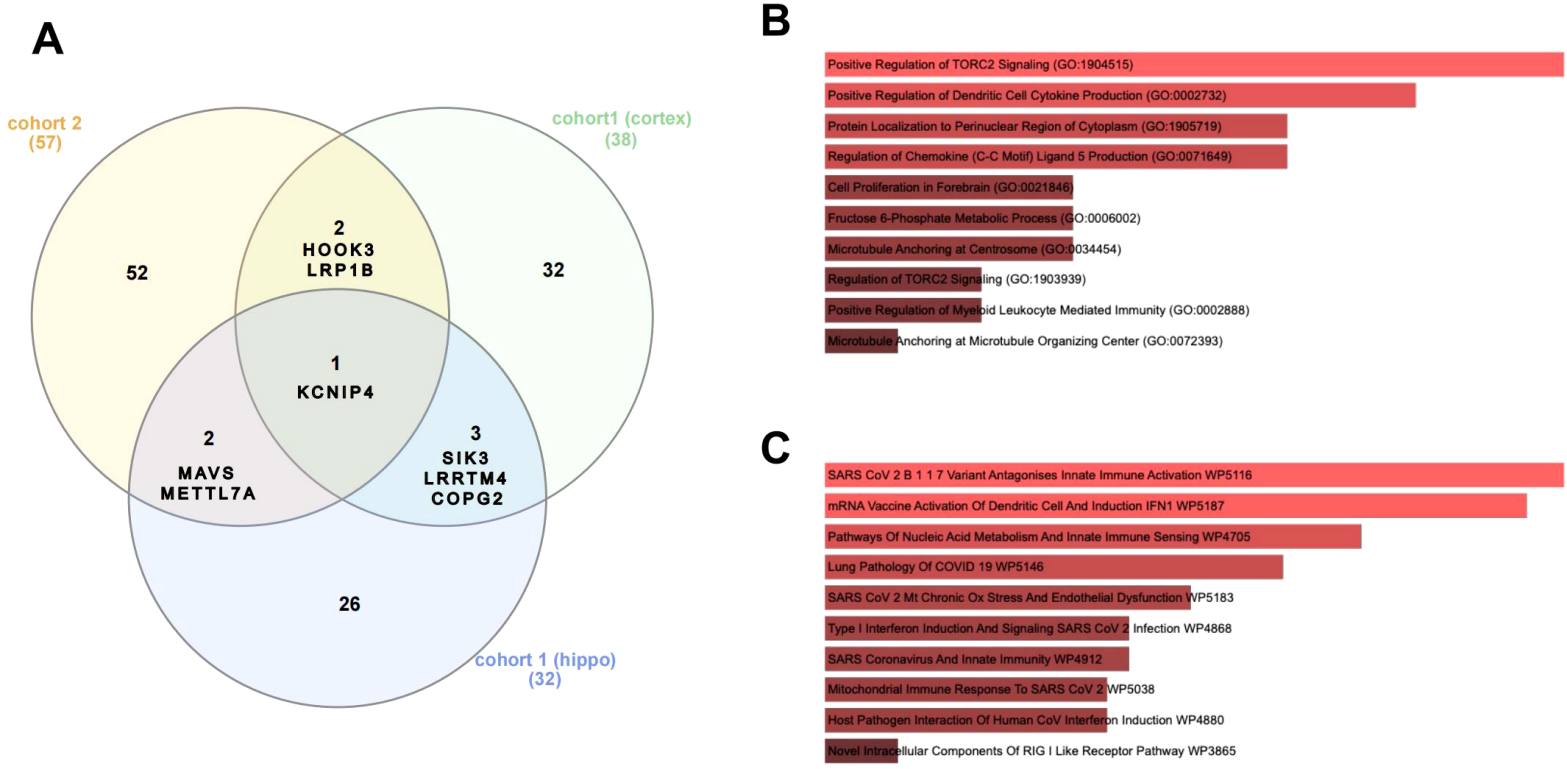
Cross-cohort and Cross-tissue analysis identified key genes with dysregulated RNA editing in the brains of sepsis patients. **(A)** Comparison of the differentially edited genes in the hippocampus and the prefrontal cortex samples of the discovery cohort (cohort 1) and the brain samples of cohort 2. Barplots show GO BP **(B)** and Wikipathway **(C)** enrichment of the eight key genes with cross-cohort and cross-tissue dysregulated RNA editing between sepsis and controls. GO, gene ontology; BP, biological process.

To further evaluate the key differentially edited genes in the brain, we compared differentially edited brain genes between different brain regions or sepsis cohorts (**Figure 4A**). Our results showed that four genes, including Potassium Voltage-Gated Channel Interacting Protein 4 (*KCNIP4*), *SIK3*, *COPG2*, and Leucine Rich Repeat Transmembrane Neuronal 4 (*LRRTM4*), were differentially edited in both the hippocampus and the prefrontal cortex. Three genes, including *KCNIP*, Mitochondrial Antiviral Signaling Protein (*MAVS*), and Thiol Methyltransferase 1A (TMT1A, also known as Methyltransferase-Like Protein 7A, *METTL7A*), were differentially edited in both the replication cohort and the hippocampus of the discovery cohort, whereas three genes, including *KCNIP*, Hook Microtubule Tethering Protein 3 (*HOOK3*), and LDL Receptor Related Protein 1B (*LRP1B*), were differentially edited in both the replication cohort and the prefrontal cortex of the discovery cohort. Notably, *KCNIP* was differentially edited in both brain regions and both cohorts. GO and Wikipathway analyses of these eight key genes suggested that they were mainly enriched in the regulation of TORC2 signaling and innate immunity, including cytokine and chemokine production (**Figures 4B, C**).

#### Inflammation altered ADAR-mediated RNA editing and expression of MAVS

**Figure 5:**
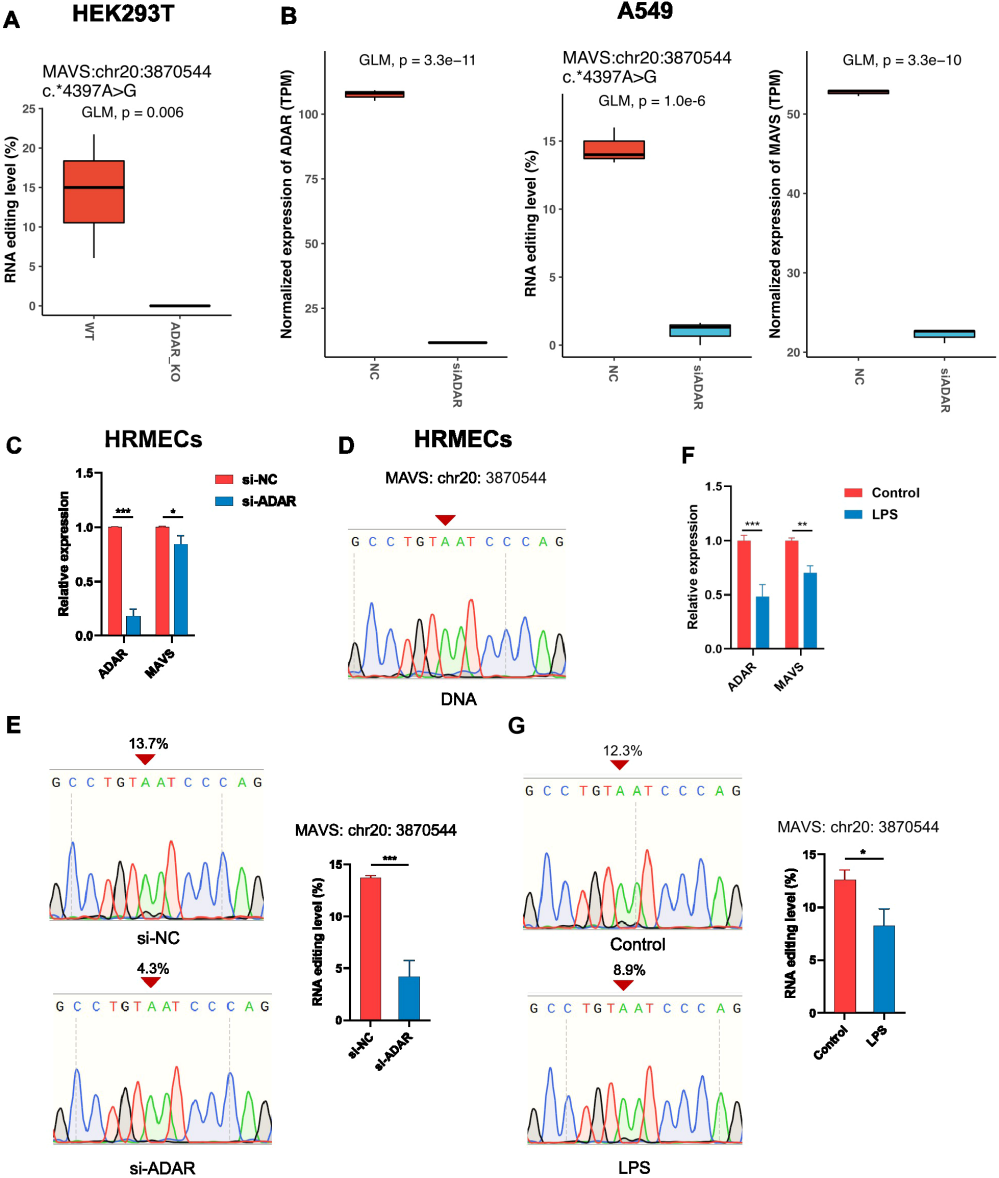
Validation of ADAR-mediated RNA editing in *MAVS* in cells. **(A)** The RNA editing levels in the 3’-UTR of *MAVS* (MAVS:chr20:3870544) in wildtype (WT) and *ADAR*-knockout (ADAR_KO) HEK293T cells. **(B)** The expression levels of *ADAR* and the expression and editing (MAVS:chr20:3870544) levels of *MAVS* in human A549 lung cancer cells treated with scramble (NC) and ADAR siRNAs. **(C)** The expression levels of *ADAR* and *MAVS* measured by qPCR in HRMECs treated with scramble (NC) and *ADAR* siRNAs. **(D)** Sanger sequencing of the MAVS DNA region. Sanger sequencing **(E)** and its quantification of the *MAVS* editing level (MAVS:chr20:3870544) in HRMECs treated with scramble (NC) and *ADAR* siRNAs. The expression level **(F)** of *ADAR* and *MASV* measured by qPCR and the Sanger sequencing of *MAVS* RNA editing (MAVS:chr20:3870544) and its quantification **(G)** in naïve and LPS-treated (2 hours) HRMECs. GLM, generalized likelihood ratio test. *P < 0.05, **P < 0.01; ***P < 0.001, calculated by student’s t test.

To further explore the functional impact of sepsis-associated differential brain RNA editing, RNA editing in *MAVS* was investigated across multiple cell lines. The differential editing sites in *MAVS* 3’-UTR (MAVS:chr20:3870544 and MAVS:chr20:3872327) in the sepsis cohorts were detected in HEK293T and A549 cells (**Figures 5A, B**). Moreover, knockout of *ADAR* substantially abolished the RNA editing at MAVS:chr20:3870544 in HEK293T cells (**Figure 5A**). Likewise, knockdown of *ADAR* downregulated RNA editing at MAVS:chr20:3870544 in A549 cells (**Figure 5B**). Moreover, knockout or knockdown of *ADAR* significantly altered the expression of *MAVS* in A549 cells, suggesting a potential regulatory role of the RNA editing at MAVS:chr20:3870544 (**Figure 5B**). Our previous reports have shown that changes in A-to-I RNA editing activity in cerebral vessels and vascular endothelial cells could be involved in acute neuroinflammation during SAE. Therefore, the potential ADAR-mediated RNA editing of *MAVS* and its expression were then analyzed in HRMECs. Knockdown of *ADAR* using siRNA substantially downregulated *MAVS* expression (**Figure 5C**) and its RNA editing at MAVS:chr20:3870544 in HRMECs (**Figures 5D, E**). To further validate the relationship between inflammation and ADAR-mediated RNA editing of MAVS and its expression, HRMECs were stimulated with LPS for 2 hours, resulting in significantly reduced RNA editing and MAVS expression levels, as confirmed by Sanger sequencing and qPCR (**Figures 5F, G**). Notably, the RNA level of *MAVS* positively correlated with its proteinlevel in the brain tissues in the cProSite database (**Supplementary Figure S2**). These findings thus validated the ADAR-mediated RNA editing in *MAVS*, suggesting its role in regulating *MAVS* expression during inflammation.

#### Positive correlation between ADAR and MAVS expression in the human hippocampus and prefrontal cortex

**Figure 6:**
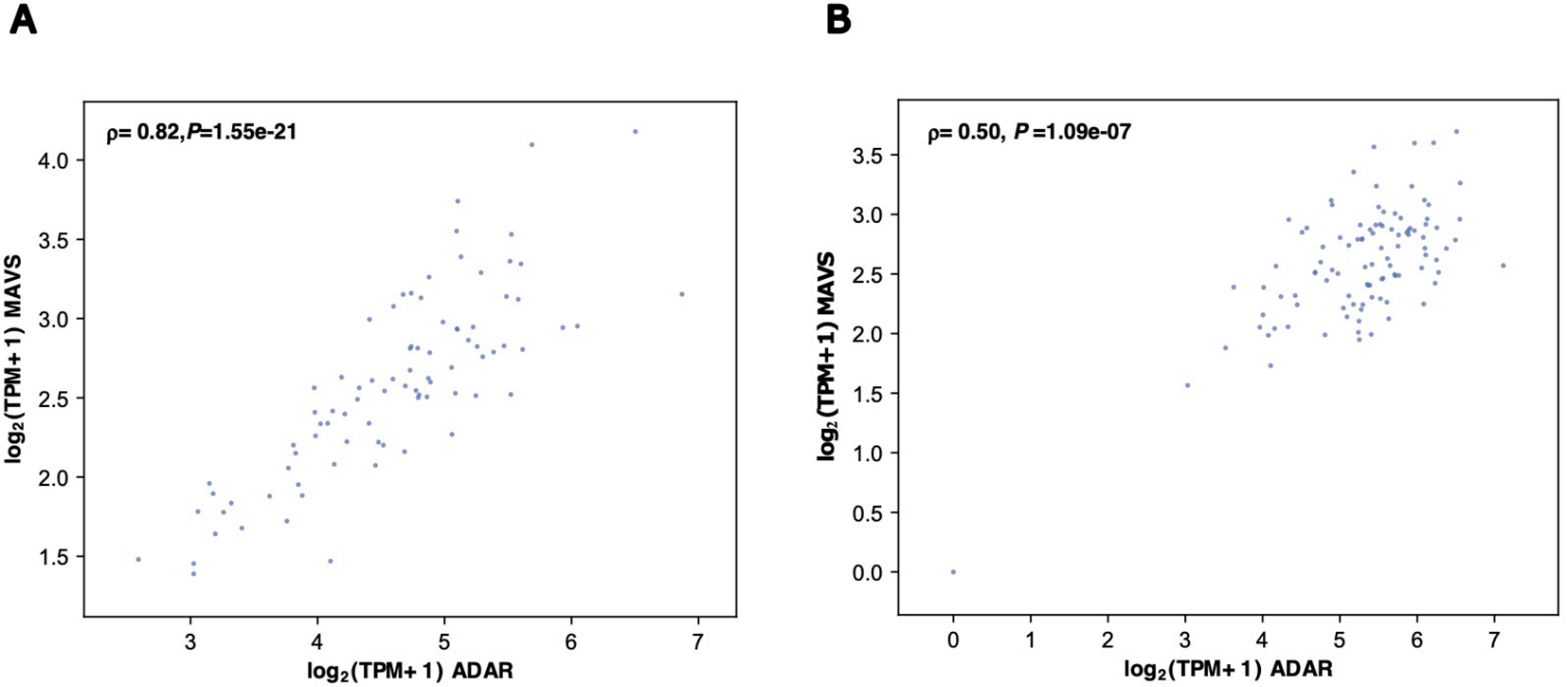
Correlation between *ADAR* and *MAVS* expression in the human hippocampus and prefrontal cortex regions in the GTEx dataset. The scatter plots are shown for the hippocampus **(A)** and prefrontal cortex **(B)**. Correlation coefficients (ρ) and *P*-values were calculated using the Spearman method.

To further explore the potential regulatory effects of *ADAR* on *MAVS* expression, the correlation between both genes was evaluated in the GTX dataset. Spearman correlation analysis showed that *ADAR* expression positively correlated with *MAVS* expression in both the human hippocampus (ρ = 0.88, *P* = 1.09×10^-21^) and prefrontal cortex (ρ = 0.5, *P* = 1.09×10^-7^) (**Figures 6A, B**). Such findings were consistent with a regulatory role for ADAR-mediated RNA editing in *MAVS* expression.

## Discussion

Our previous reports have demonstrated dynamic brain A-to-I RNA editing changes associated with SAE in both patients and mouse models (19–21). However, before our current study, A-to-I RNA editing profiles have not been thoroughly characterized in different brain regions of sepsis patients. Our epitranscriptome-wide analysis uncovered varied dysregulation of RNA editing in the hippocampus and prefrontal cortex of these patients.

Our findings showed region-specific dysregulation of RNA editing in the brains of sepsis patients. RNA editing is developmentally regulated and may be stimulus-responsive, particularly in diseases, depending on the tissue or cell types (12, 13). For instance, the brain exhibits high editing levels in neurotransmission-related genes, such as GRIA2, which affects AMPA receptor function (34, 35). Editing levels of several brain-specific transcripts increase during brain development (12). Moreover, inflammation can also induce editing in immune cells (18). In our current study, sepsis patients exhibited a substantial reduction in the average RNA editing level in the hippocampus but not in the prefrontal cortex, suggesting a difference in overall RNA editing activity between the brain regions in the disease. Additionally, the sites and genes with differential editing exhibited differences between the two brain regions. These findings emphasize the complexity of RNA editing dysregulation in different brain regions of sepsis patients, which is crucial for understanding the epitranscriptomic abnormalities associated with the disease.

Our results highlighted key genes with cross-validated dysregulated RNA editing in the brains of sepsis patients, particularly in the hippocampus, indicating underlying common epitranscriptomic changes, with notable differences between the two brain regions. Such a region difference could be attributable to the observed differences in ADAR expression between them. Such findings could align with differences in region-specific vulnerability to sepsis previously reported for the two brain regions. The hippocampus exhibits greater vulnerability to sepsis-induced inflammation than the prefrontal cortex, characterized by markedly elevated pro-inflammatory cytokines and severe metabolic disruption, whereas the prefrontal cortex exhibits distinct microglial activation patterns, with cellular proliferation and enhanced GABAergic protective responses (36–38).

The *MAVS* gene encodes an intermediary protein necessary for the virus-triggered beta interferon signaling pathways, which are pivotal in antiviral innate immunity. It activates inflammatory responses upon detecting viral RNA (39). Beyond viral infections, MAVS has been implicated in the immune response during sepsis, and dysregulation of MAVS signaling could contribute to the excessive inflammation characteristic of sepsis (40). Our results showed that ADAR-mediated RNA editing in *MAVS* was substantially dysregulated in the brain, especially in the hippocampus, during sepsis. LPS stimulation results suggested that inflammation could influence the RNA editing level of *MAVS* 3 ‘UTR, potentially regulating MAVS expression in turn, in vascular endothelial cells. These findings were consistent with our previous reports on dysregulated RNA editing associated with cerebrovascular dysfunction during acute inflammation in a mouse model. MAVS signaling has been reported to be required for virus sensing, full microglial activation, and immune defense in the CNS during viral infection (41). Therefore, these findings suggest a vital role for ADAR-mediated *MAVS* RNA editing in regulating MAVS signaling during SAE development. It remains undetermined whether the altered MAVS RNA editing affects its protein level, its RNA localization, or downstream signaling, which warrants further study. In addition, *METTL7A* showed similar cross-cohort dysregulated RNA editing in the brains of sepsis patients. *METTL7A* or *TMT1A* encodes a member of thiol S-methyltransferases, and has recently been suggested to be a new epitranscriptomic enzyme with mRNA m6A methyltransferase activity, which can methylate the N6 position of adenosine residues in long non-coding RNAs (42). Our results thus suggest a potential cross-talk between A-to-I RNA editing and m6A in sepsis-associated brain dysfunctions.

Our findings also suggested a potential role for RBPs in RNA editing dysregulation in the brains of sepsis patients. HNRNP0 was predicted to overlap most frequently with the differentially edited RNA sites across both brain regions in sepsis patients, and its expression was strongly correlated with the average RNA editing level. HNRNP0 is an RBP involved in various cellular processes, including mRNA translation and regulation, as well as diseases, including cancer and neurodegenerative diseases (43, 44). These findings warranted further study into the role of HNRNP0 in RNA editing and sepsis.

Our study has several limitations that warrant further investigation. First, validation of the identified editing changes in larger, well-characterized cohorts will be needed to confirm the robustness and generalizability of our findings. Second, our results are based on bulk tissue RNA-Seq and *in vitro* cell experiments, precluding causal inference and limiting insight into cell–type–specific editing dynamics. Future work, including *in vivo* and *in vitro* manipulation of RNA editing at specific MAVS and other candidate sites, will be necessary to establish causal links between ADAR-mediated editing, MAVS signaling, and SAE. In addition, our results did not distinguish the contributions of individual ADAR isoforms. Systematic dissection of ADAR isoforms across distinct brain cell populations during systemic inflammation will be critical for refining the mechanistic framework emerging from our data.

In conclusion, our current study revealed diverse dysregulation of RNA editing in the hippocampus and prefrontal cortex of sepsis patients. Moreover, the cross-cohort analysis and functional validation underscore the potential role of ADAR-mediated RNA editing and the innate immune defense gene *MAVS* as a key target in the septic brain.

## Data Availability

The original contributions presented in the study are included in the article/**Supplementary Material**. Further inquiries can be directed to the corresponding author/s.

## Supplementary material

Supplementary Table 1 The siRNAs and primers used in the current study.

Supplementary Table 2 Identified high-confidence A-to-I RNA editing sites in the brain samples.

Supplementary Table 3 Identified high-confidence A-to-I RNA editing sites in the hippocampus samples.

Supplementary Table 4 Identified high-confidence A-to-I RNA editing sites in the prefrontal cortex samples.

Supplementary Table 5 Differential A-to-I RNA editing sites in the brain samples.

Supplementary Table 6 Differential A-to-I RNA editing sites in the hippocampus samples.

Supplementary Table 7 Differential A-to-I RNA editing sites in the prefrontal cortex samples.

Supplementary Table 8 cis-regulatory effect of differential A-to-I RNA editing on edited gene expression in all samples.

Supplementary Table 9 Cis-regulatory effect of differential A-to-I RNA editing on edited gene expression in the hippocampus samples.

Supplementary Table 10 Cis-regulatory effect of differential A-to-I RNA editing on edited gene expression in the cortex samples.

Supplementary Figure 1 Bar plot showing *ADAR* expression in hippocampal and prefrontal cortex tissues from sepsis and controls. The overall significance P-value was calculated using GLM and a likelihood ratio test, followed by Tukey’s *post-hoc* test. **P* < 0.05.

Supplementary Figure 2 Correlation between the RNA and protein levels of MAVS in the brain tissues in the cProSite database.
