# Supplementary figures and images for: Editome profiling and cross-cohort validation reveal A-to-I RNA editing dysregulation in the hippocampus and prefrontal cortex of sepsis patients
Source: Front Psychiatry. 2026 Mar 3;16:1742710. doi: 10.3389/fpsyt.2025.1742710 (PMC12993761; doi:10.3389/fpsyt.2025.1742710)

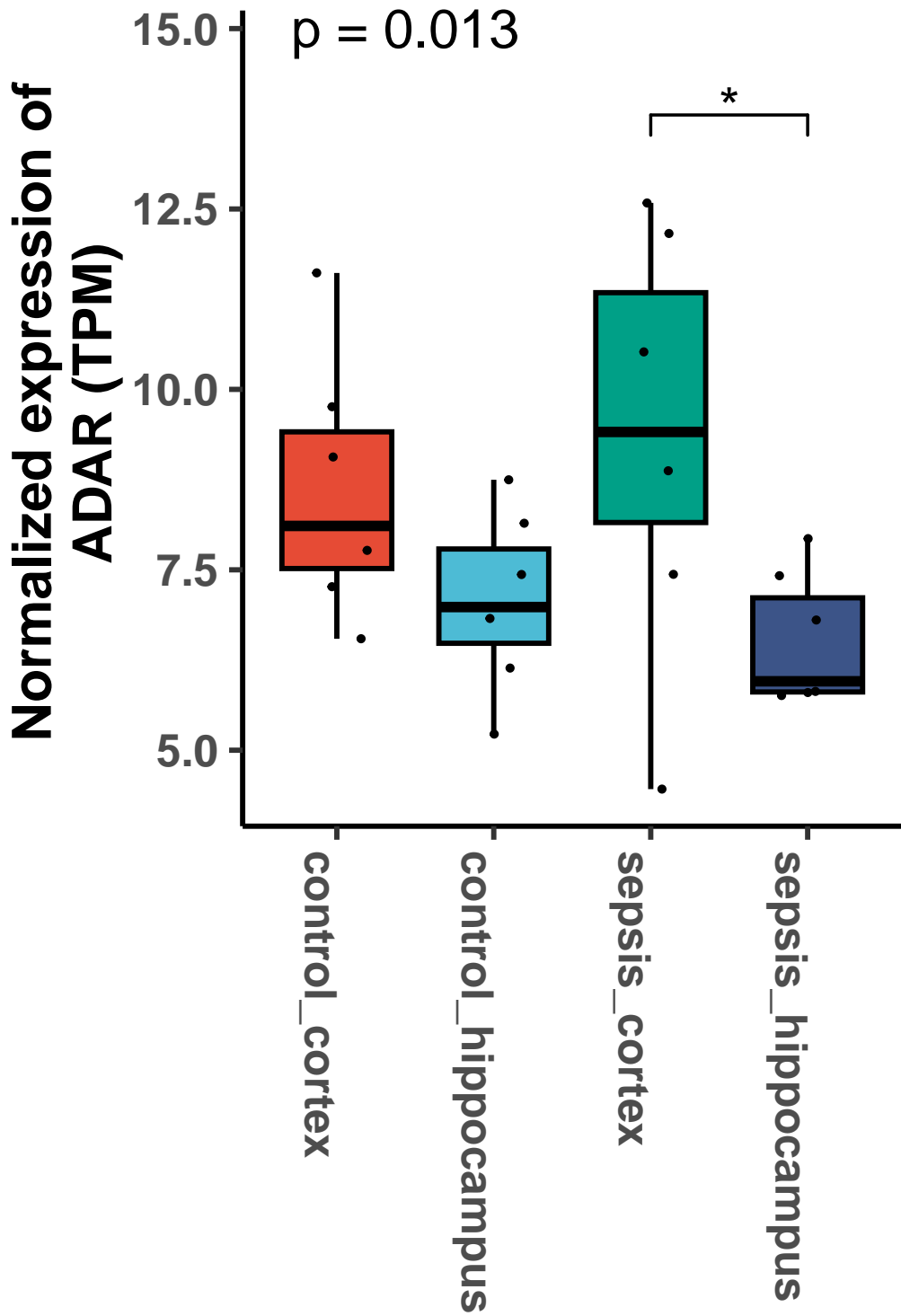

Supplement: Supplementary Figure 1 — Bar plot showing ADAR expression in hippocampal and prefrontal cortex tissues from sepsis and controls. The overall significance P-value was calculated using GLM and a likelihood ratio test, followed by Tukey’s post-hoc test. *P < 0.05. [file Image1.pdf]

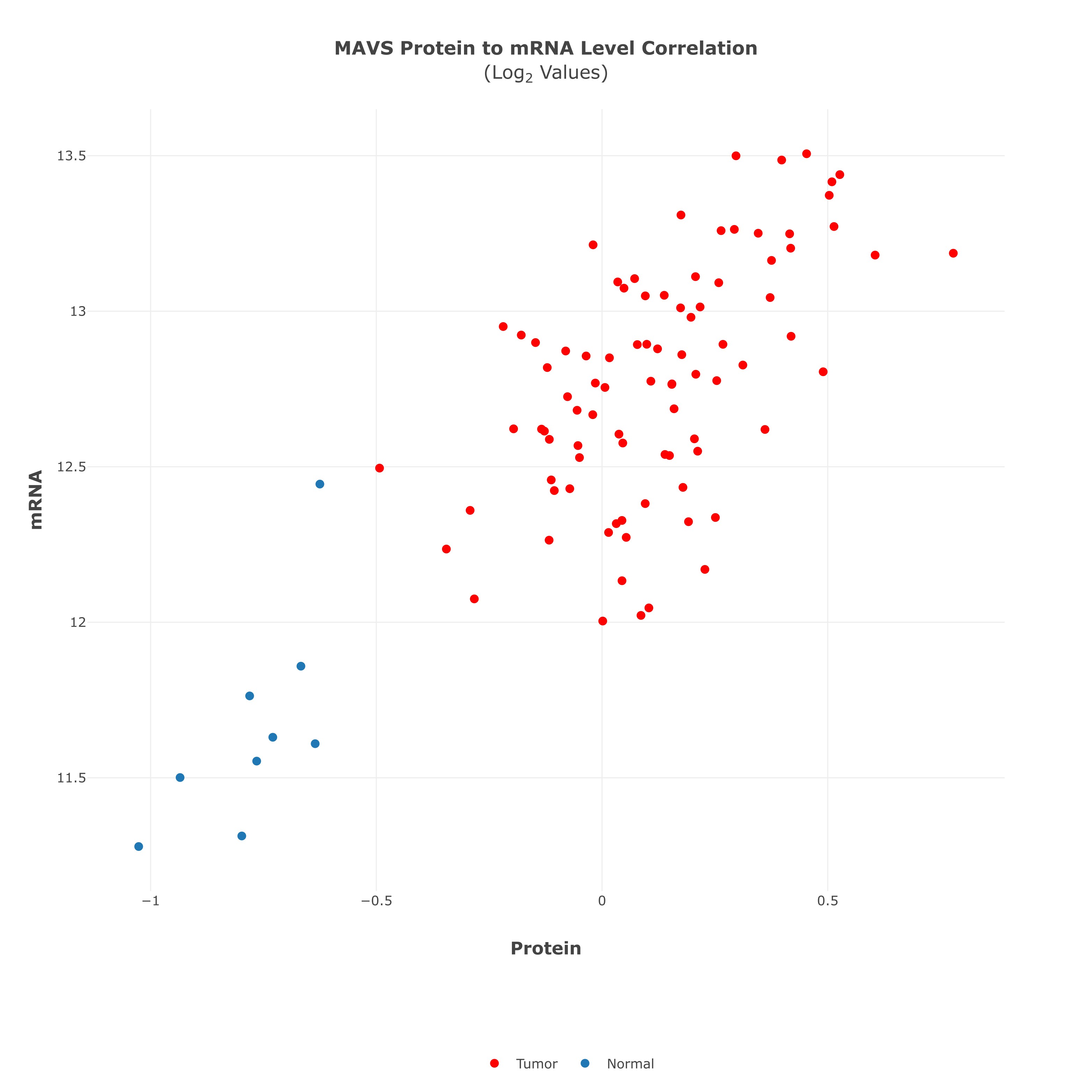

Supplement: Supplementary Figure 2 — Correlation between the RNA and protein levels of MAVS in the brain tissues in the cProSite database. [file Image2.jpeg]
